# Complete Genome Sequences of Three *Streptococcus agalactiae* Serotype Ia Isolates Obtained from Disease Outbreaks in Nile Tilapia (*Oreochromis niloticus*)

**DOI:** 10.1128/genomeA.01432-17

**Published:** 2018-01-04

**Authors:** Anita Jaglarz, Artur Gurgul, William J. Leigh, Janina Z. Costa, Kim D. Thompson

**Affiliations:** a Aquaculture Research Group, Moredun Research Institute, Bush Loan, Penicuik, United Kingdom; b Department of Genomics and Molecular Biology of Animals, The National Research Institute of Animal Production, Balice, Poland

## Abstract

This paper describes the whole-genome sequences for three *Streptococcus agalactiae* serotype Ia isolates. The isolates were recovered from the brains of clinically sick tilapia, *Oreochromis niloticus*, that were suffering from streptococcosis. One isolate was from tilapia in the United States and the other two from fish in China.

## GENOME ANNOUNCEMENT

Streptococcosis has been highlighted as a major disease problem for the tilapia aquaculture industry (1, 2). Three *Streptococcus agalactiae* isolates were recovered from the brains of diseased Nile tilapia, *Oreochromis niloticus*, during streptococcosis outbreaks on tilapia farms in the United States and China. Here, we analyzed three strains of serotype Ia belonging to sequence type (ST) 7 and clonal complex (CC) 7. The isolates were characterized by molecular serotyping (3) and multilocus sequence typing (MLST) (4). For genomic sequencing of these isolates, a single colony of the bacteria was cultured for 24 h in tryptone soya broth (Oxoid, Basingstoke, UK) at 28°C, and genomic DNA was obtained from the cultured bacteria using a DNeasy Blood & Tissue kit (Qiagen, Manchester, UK) according to the manufacturer’s protocol for Gram-positive bacteria.

Sequencing was performed using an Illumina (San Diego, CA) MiSeq System, employing a 250-bp paired-end read of the libraries (550-bp average insert size) prepared with a TruSeq nano DNA library preparation kit (Illumina, San Diego, CA). *De novo* assembly of the reads was performed with SPAdes v. 3.5.0 software (5), and the assembly quality was evaluated using QUAST software (6). The resulting contigs (>200 nucleotides [nt]) were annotated using the NCBI Prokaryotic Genome Annotation Pipeline, which includes prediction of protein-coding genes, as well as other functional genome units, such as structural RNAs, tRNAs, small RNAs, pseudogenes, control regions, direct and inverted repeats, insertion sequences, transposons, and other mobile elements.

The genomes of *S. agalactiae* isolates 14-66, 14-107, and 14-119 (2,039,286, 2,126,695, and 2,002,520 bp) were distributed in 79, 106, and 51 contigs and based on average *de novo* assembly coverage of 193×, 227×, and 154×, respectively. The G+C genome content was 35.4% for isolates 14-66 and 14-119 and 35.5% for isolate 14-107. The genome of isolate 14-66 contained a total of 2,134 genes, including 1,983 coding genes, 88 RNA genes (80 tRNAs), and 65 pseudogenes. The 14-107 genome contained 2,234 genes, including 2,076 coding genes, 86 RNA genes (80 tRNAs), and 72 pseudogenes. The genome of 14-119 comprised a total of 2,078 genes with 1,945 coding genes and 83 RNA genes, including 77 tRNAs and 50 pseudogenes. In addition, each genome was found to have a putative clustered regularly interspaced short palindromic repeat (CRISPR).

### Accession number(s).

The genome sequences of *S. agalactiae* strains 14-66, 14-107, and 14-119 have been deposited in the GenBank database under the accession numbers NKQA00000000, NKPZ00000000, and NKPY00000000, respectively.
